# Ferroptosis: Therapeutic Potential and Strategies in Non-Small Cell Lung Cancer

**DOI:** 10.3390/biology14050545

**Published:** 2025-05-14

**Authors:** Ying Lei, Shuxia Jiang, Chengyu Kong, Ping Pang, Hongli Shan

**Affiliations:** Shanghai Frontiers Science Research Center for Druggability of Cardiovascular Noncoding RNA, Institute for Frontier Medical Technology, School of Chemistry and Chemical Engineering, Shanghai University of Engineering Science, Shanghai 201620, China; leiying@sues.edu.cn (Y.L.); kongchengyu@sues.edu.cn (C.K.); pangping@sues.edu.cn (P.P.)

**Keywords:** ferroptosis, characteristics, mechanism, genetic regulation, therapeutic strategies, NSCLC

## Abstract

Non-small cell lung cancer (NSCLC) is the most common subtype of lung cancer and a leading cause of cancer-related morbidity and mortality worldwide. Despite advancements in therapeutic strategies, the prognosis for NSCLC patients remains unfavorable. The effective treatment of NSCLC remains challenging due to its aggressive metastatic and invasive properties. Therefore, there is an urgent need to explore novel treatment strategies. In recent years, different from apoptosis and necrosis, ferroptosis has garnered increasing attention since its initial identification in 2012. It is increasingly recognized as a key factor in the development and progression of various cancers. In this review, we summarize the distinctive morphological and biochemical characteristics of ferroptosis and its regulatory mechanisms. Furthermore, we discuss the genetic regulation of ferroptosis in NSCLC, highlighting key biomarkers that may serve as potential therapeutic targets. We also evaluate emerging therapeutic strategies targeting ferroptosis, including gene therapy, natural compounds, chemical agents, combination therapies, and nanoparticle-based approaches. Based on current evidence, the limitations and future prospects of ferroptosis-based therapies for NSCLC are discussed. This review aims to provide novel insights into the potential of ferroptosis-based therapies for NSCLC and its implications for the development of novel treatments.

## Simple Summary

Non-small cell lung cancer (NSCLC) remains a major clinical challenge due to its high metastatic potential and limited responsiveness to the current treatment options. Ferroptosis, an iron-dependent form of programmed cell death, has emerged as a potential therapeutic mechanism distinct from traditional cell death pathways. This review provides a comprehensive overview of the morphological and biochemical features of ferroptosis, elucidates its unique regulatory networks in NSCLC, and proposes key genetic markers associated with its regulation. We also assess the potential of ferroptosis-based interventions, including gene therapy, natural and synthetic compounds, combination treatments, and nanoparticle-mediated delivery systems. By critically analyzing recent advances and the existing research gaps, this review highlights the relevance of ferroptosis as an innovative therapeutic strategy for NSCLC, with the potential to improve treatment efficacy and patient survival outcomes, ultimately contributing to the advancement of NSCLC therapy.

## 1. Introduction

**Figure 1:**
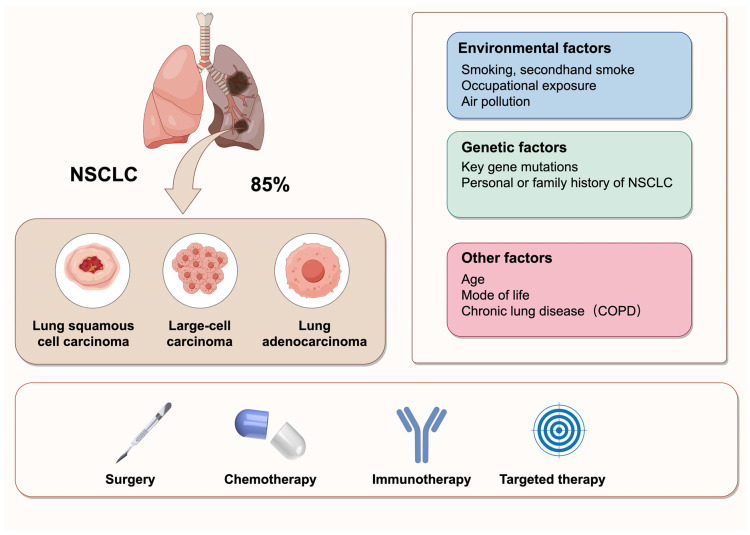
Classification, influencing factors, and treatment strategies of NSCLC. NSCLC is the most prevalent type of lung cancer, accounting for 85% of all cases. It primarily comprises three histological subtypes: lung squamous cell carcinoma, large-cell carcinoma, and lung adenocarcinoma. The pathogenesis of NSCLC is multifactorial, involving environmental exposures, genetic predispositions, and other contributing factors. The current treatment strategies for NSCLC include surgery, chemotherapy, immunotherapy, and targeted therapy.

Cancer is a major global health concern, with an estimated 2.2 million new cases in 2024. Lung cancer remains the leading cause of cancer-related mortality worldwide, responsible for approximately 1.8 million deaths in 2024 [1]. Non-small cell lung cancer (NSCLC), which includes subtypes such as lung adenocarcinoma (LUAD), large cell carcinoma (LCLC), and lung squamous cell carcinoma (LUSC), accounts for approximately 85% of lung cancers [2]. NSCLC is characterized by high metastasis and invasiveness, and it is frequently diagnosed at an advanced stage. The 5-year survival rate has improved but is still lower than other cancers [1]. Despite significant advancements in treatment options, including chemotherapy, radiotherapy, immunotherapy, and surgery, the management of NSCLC remains challenging due to frequent metastasis and poor prognosis [3] (Figure 1). Thus, there is a critical need for novel and effective therapeutic strategies to improve the clinical outcomes for NSCLC patients.

Ferroptosis is an iron-dependent form of programmed cell death (PCD), characterized by iron accumulation, excessive generation of reactive oxygen species (ROS), lipid peroxidation, and mitochondrial dysfunction [4]. Unlike other types of PCD, such as necroptosis, autophagy, and apoptosis, ferroptosis displays distinct differences in both cell morphology and biochemical characteristics [5]. Ferroptosis, as an emerging therapeutic strategy, has demonstrated significant potential in addressing tumor progression and therapeutic resistance of various cancers. In lung cancer, genetic mutations or aberrant gene activation often lead to elevated intracellular oxidative stress. Compared to normal tissues, lung cancer tissues exhibit heightened sensitivity to oxidative stress due to their prolonged exposure to a high-oxygen microenvironment. The accumulation of ROS and lipid peroxides in NSCLC tissues indicates their heightened susceptibility to ferroptosis. Notably, studies have shown that 88% of NSCLC patients exhibited high expression of transferrin receptor 1 (TFR1), and 62% displayed elevated ferritin levels, contributing to intracellular iron accumulation and creating favorable conditions for the induction of ferroptosis [6,7]. In recent years, ferroptosis has attracted considerable attention as a novel therapeutic strategy for the treatment of NSCLC [8].

In this review, we summarize recent advances in the understanding of ferroptosis in NSCLC by outlining its key morphological and biochemical characteristics, elucidating the core metabolic and genetic pathways involved in its regulation, and evaluating both the current and emerging therapeutic strategies that leverage ferroptosis for NSCLC treatment. Furthermore, we discuss the limitations of the current research and highlight future directions for the development of ferroptosis-based therapies. By providing a thorough overview of the current landscape, this review aims to offer new insights into ferroptosis as a promising avenue for NSCLC therapy.

## 2. Characteristics of Ferroptosis

The characteristic cellular changes associated with ferroptosis include loss of cell membrane integrity, increased mitochondrial membrane density, mitochondrial shrinkage, and a reduction in cristae [4]. These morphological features distinguish ferroptosis from apoptosis, necroptosis, and autophagy (Table 1).

## 3. Mechanisms That Promote Ferroptosis

**Figure 2:**
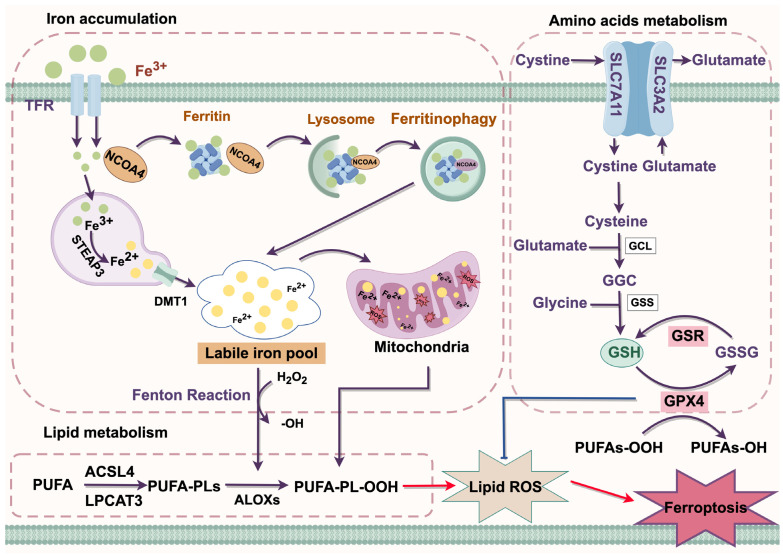
Molecular mechanism of ferroptosis. ACSL4, Acyl-CoA synthetase long-chain family member 4; ALOX, arachidonate lipoxygenase; Cys, cysteine; DMT1, divalent metal transporter 1; GGC, glutamate/glutamate/cysteine; Glu, glutamate; GPX4, glutathione peroxidase 4; GSR, glutathionedisulfide reductase; GSSG, oxidized glutathione; GSH, glutathione; Lipid ROS, lipid reactive oxygen species; LPCAT3, lysophosphatidylcholine acyltransferase 3; NAPD⁺, Nicotinamide adenine dinucleotide phosphate (oxidized form); NAPDH, Nicotinamide adenine dinucleotide phosphate (reduced form); PE-PUFA, Phosphatidylethanolamine-polyunsaturated fatty acid; PE-PUFA-OH, Hydroperoxy polyunsaturated fatty acid; PE-PUFA-OOH, Peroxy polyunsaturated fatty acid; PUFA, polyunsaturated fatty acid; SLC7A11, solute carrier family 7 member a11; STEAP3, prostate transmembrane epithelial antigen 3; TFR, transferrin receptor. Red arrow = activation; Blue arrow = inhibition; Purple arrow = positive effects.

The complex interplay between iron, lipid, and amino acid metabolism plays a crucial role in ferroptosis. Mechanistically, ferroptosis is driven by the accumulation of intracellular iron and lipid peroxides [9] (Figure 2).

### 3.1. Iron Accumulation

Iron functions as a crucial cofactor involved in various cellular metabolic and physiological processes [10]. In circulation, it primarily exists in the form of Fe^3+^, which binds with transferrin (TF) to form the TF-Fe^3+^ complex. This complex is recognized by the transferrin receptor protein (TFR) and subsequently internalized into cells through the classical receptor-mediated endocytosis system. Within the acidic environment of endosomes, Fe^3+^ is released from TF and subsequently reduced to Fe^2+^ by prostate transmembrane epithelial antigen 3 (STEAP3). Fe^2+^ is then released into the cytoplasm via the divalent metal transporter 1 (DMT1), where it primarily exists as a labile iron pool (LIP) [4]. Excessive accumulation of LIP triggers ferroptosis by generating ROS through the Fenton reaction, which in turn promotes lipid peroxidation [11].

Ferritinophagy is a form of selective autophagy that enhances sensitivity to ferroptosis by regulating the homeostasis of the cellular LIP [12]. Under normal conditions, excess iron is mostly stored in ferritin in the form of redox inactivity. Nuclear receptor coactivator 4 (NCOA4) was identified as a ferritin receptor that mediates ferritin degradation through the formation of a lysosome-targeted ferritin complex, thereby releasing excess iron and positively regulating ferroptosis [13].

### 3.2. Lipid Metabolism

Lipid peroxidation occurs through three stages: initiation, propagation, and termination, which are precisely regulated by Acyl-CoA synthase long-chain family member 4 (ACSL4), arachidonate-15-Lipoxygenase (ALOX15), and lysophosphatidylcholine acyltransferase 3 (LPCAT3) [14]. Polyunsaturated fatty acids (PUFAs) are major components of cellular membranes, and phosphatidyl ethanolamine (PE) containing arachidonic acid (AA) or its derivative adrenic acid (AdA) are key phospholipids that drive ferroptosis among the phospholipids associated with PUFAs [15]. Under the catalysis of ACSL4, AA or AdA is esterified to form AA-CoA or AdA-CoA. Subsequently, these acyl-CoA derivatives are then incorporated into membrane phospholipids to generate AA-PE or AdA-PE by LPCAT3, and finally oxidized by ALOX5 to generate PE-AdA-OOH and PE-AA-OH. The accumulation of these peroxidized phospholipids contributes to the initiation of ferroptosis [16,17].

### 3.3. Amino Acids Metabolism

The system X_c_^−^ is a heterodimeric amino acid transporter composed of solute carrier family 3 member 2 (SLC3A2) and solute carrier family 7 member 11 (SLC7A11), mediating the exchange of glutamate and cystine between the intracellular and extracellular at a molar ratio of 1:1. The ingested cystine is reduced intracellularly to cysteine, which is then utilized in the synthesis of glutathione (GSH) [18]. GSH plays a crucial role in ferroptosis resistance by eliminating toxic lipid peroxides through glutathione peroxidase 4 (GPX4) [19]. Inhibition of the system X_c_^−^ activity reduces the synthesis of GSH, leading to the accumulation of lipid peroxidation and ultimately triggering ferroptosis [20].

## 4. Genetic Regulation of Ferroptosis in NSCLC

Since the discovery of ferroptosis in 2012, several genes have been identified as critical biomarkers of this process [20]. Recent studies have highlighted the key genes involved in regulating ferroptosis in NSCLC, suggesting potential therapeutic targets for more effective cancer treatment. The specific regulatory mechanisms are detailed in Table 2.

### 4.1. SLC7A11

The regulation of SLC7A11, a critical component of X_c_^−^, tightly governs the balance between ferroptosis inhibition and promotion in NSCLC cells. Analyses of The Cancer Genome Atlas (TCGA) and Gene Expression Omnibus (GEO) databases revealed significantly elevated expression of SLC7A11 in tumor tissues compared to normal tissues. Moreover, the suppression of SLC7A11 weakened the proliferation and viability in H358 cells, further supporting the suggestion that high SLC7A11 levels are indicative of a poor prognosis in NSCLC patients [21]. A subunit of the spliceosome factor 3B 1 (SF3B1) has been reported to be overexpressed in NSCLC tissues compared to adjacent normal tissues and inhibited ferroptosis by upregulating SLC7A11, thereby promoting tumorigenesis and progression in lung LUAD through the enhanced proliferation, migration, and invasion of LUAD cells [22]. Conversely, the tripartite motif-containing 3 (TRIM3) expression was downregulated in clinical NSCLC samples and exhibited a negative correlation with xCT protein levels. The overexpression of TRIM3 has been shown to promote ferroptosis in NSCLC by directly interacting with SLC7A11/xCT via its NHL domain, inducing K11-linked ubiquitination and the subsequent proteasomal degradation of SLC7A11 [23]. Another study revealed that the lysosome-associated protein transmembrane 4B (LAPTM4B) protected against ferroptosis in NSCLC by inhibiting ubiquitin–proteasome degradation of the cystine/glutamate antiporter SLC7A11, a process mediated by NEDD4L/ZRANB1 both in vivo and in vitro [24]. HOXB9, a potential transcriptional regulator of SLC7A11, was upregulated in NSCLC tissues and functions as a tumor promoter in cancer progression. The knockdown of HOXB9 suppressed cell proliferation and migration, whereas its overexpression enhanced these processes in NSCLC cells. Notably, the acetyltransferase p300/CBP-associated factor (PCAF) interacts with HOXB9 and mediates its acetylation. The knockdown of PCAF enhanced the binding affinity of HOXB9 to the SLC7A11 promoter, whereas PCAF overexpression inhibited HOXB9 binding, thereby downregulating SLC7A11 expression and suppressing the growth and progression of NSCLC [21]. Additionally, ALKBH5 was prominently downregulated in NSCLC patients, and its upregulation has been shown to inhibit tumor progression by promoting ferroptosis through the suppression of SLC7A11 expression. Mechanistically, ALKBH5 downregulated SLC7A11 expression by lowering m6A modification [25]. These findings suggest that targeting the epigenetic regulation of ferroptosis holds significant therapeutic potential for the treatment of NSCLC, whereas the key epigenetic regulators involved in the modulation of ferroptosis in NSCLC cells remain to be further investigated.

### 4.2. GPX4

Previous studies have shown that the downregulation of GPX4 promoted ferroptosis by increasing hydroperoxide levels or reactivating the lipoxygenase pathway, which is accompanied by the production of substantial amounts of ROS [26]. It has also been demonstrated that peroxisome proliferator-activated receptor (PPAR) bound to the peroxisome proliferator response element (PPRE) element within intron 3 to activate GPX4 and downregulated TFR expression, alleviating ferroptosis induced by iron overload [27]. Furthermore, drug-induced lactate conferred ferroptosis resistance in the NSCLC-dependent p38-SGK1 pathway by attenuating the NEDD4L/GPX4-mediated ubiquitination and degradation of GPX4 [28]. β-elemene was demonstrated to trigger ferroptosis via the TFEB-mediated lysosomal degradation of GPX4 in EGFR wild-type NSCLC [29]. Another study suggested that METTL14 promoted NSCLC progression by mediating the m6A modification of GPX4 through IGF2BP1, enhancing the stability and expression of GPX4 mRNA [30]. Ni et al. [31] demonstrated that the inhibition of mTORC1 overcame NSCLC resistance to lapatinib by promoting ferroptosis via the downregulation of GPX4 and the enhancement of intracellular ROS, MDA, and Fe^2+^ levels. Additionally, targeting GPX4 may represent a novel strategy to improve the anti-tumor effects of lapatinib. The knockdown of Notch3 induced ferroptosis in NSCLC cells by increasing intracellular ROS, lipid peroxidation, and Fe^2+^ levels while downregulating GPX4 expression, which was reversed by ferroptosis inhibitors ferstatin-1 and liproxstatin-1 [32].

### 4.3. NRF2

Nuclear factor erythroid 2-related factor 2 (NRF2) is one of the most extensively studied transcription factors in ferroptosis that regulates ferroptosis through multiple pathways [33,34]. Trabectedin has been reported to upregulate iron, ROS, and lipid peroxidation by enhancing the HIF-1α/IRP1/TFR1 axis and inhibiting the KEAP1/NRF2/GPX4 pathway, leading to ferroptosis in NSCLC cells [35]. Chen et al. [36] discovered that NSUN2 promoted the stability of NRF2 mRNA through m5C modification, thereby enhancing NRF2 protein expression, reducing lipid peroxidation, and inhibiting ferroptosis. In addition, targeting NRF2 was demonstrated to reverse the radioresistance of NSCLC by inducing mitochondrial dysfunction and promoting ferritinophagy through the upregulation of PHKG2 expression [37]. APOC1 mediated the conversion of M2 macrophages into M1 macrophages through the NRF2/HO-1 pathway, reducing the sensitivity of NSCLC for anti-PD-1 immunotherapy by promoting ferroptosis [38]. The inhibition of NRF2 has been demonstrated to enhance the sensitivity of NSCLC cells to cystine deprivation-induced ferroptosis by upregulating FOCAD expression and activating the FAK pathway [39].

### 4.4. p53

Over the past decade, accumulating evidence has highlighted the crucial role of p53 in anti-tumor by regulating ferroptosis through multiple pathways [40]. Under oxidative stress, p53 suppressed cystine uptake by downregulating the expression of SLC7A11 and inhibiting GPX4 activity, sensitizing NSCLC cells to ferroptosis [41,42,43]. Previous studies have shown that the disulfide/copper complexes activated the efficacy of the adavosertib in p53-deficient NSCLC through ferroptosis [44]. The Qingrehuoxue formula has been found to inhibit NSCLC tumorigenesis by activating both ferroptosis and apoptosis through the p53 and GSK-3/NRF2 signaling pathways [45]. Silencing the m6A methyltransferase KIAA1429 suppressed NSCLC progression by promoting ferroptosis through the activation of the p53 signaling pathway [46]. Knockdown HEATR1 induced ferroptosis by activating the p53/SAT1/ALOX15 axis with the excessive generation of ROS, suppressing cisplatin resistance in NSCLC [47].

These studies highlight the complex regulation of ferroptosis in NSCLC through key proteins, including SLC7A11, GPX4, NRF2, and p53, which together form an intricate network that regulates the balance between cell survival and death. Targeting these pathways, either individually or in combination, could enhance the efficacy of the existing therapies and offer novel strategies to overcome resistance in NSCLC. Furthermore, epigenetic and post-translational modifications present promising therapeutic opportunities to improve NSCLC treatment outcomes. However, due to the non-specificity of ferroptosis-related targets and the complexity of the upstream regulatory networks, further studies are required to identify the specific targets and elucidate their corresponding regulatory mechanisms, enabling the precise modulation of ferroptosis in NSCLC.

**Table 2:** Genetic regulation of ferroptosis in NSCLC.

Gene	Mechanism	Effect on Ferroptosis	References
SLC7A11	SPTBN2 interacts with SLC7A11 via its CH domain, links it to the motor protein Arp1, and promotes SLC7A11 membrane localization	↓	[48]
	LAPTM4B inhibits the ubiquitin/proteasomal degradation of SLC7A11 through NEDD4L/ZRANB1	↓	[24]
	Direct interaction with SLC7A11/xCT via its NHL domain leads to the ubiquitination of K11 junctions and subsequent proteasome degradation	↑	[23]
	CAP inhibits the expression of SLC7A11 by activating the PCAF-mediated acetylation of HOXB9	↑	[21]
	Downregulated SLC7A11 by reducing m6A modification	↑	[25]
	SF3B1 promotes the expression of SLC7A11	↓	[22]
GPX4	PPARα promotes the expression of GPX4	↓	[27]
	Lactate through the p38-SGK1 pathway attenuates NEDD4L/GPX4-mediated ubiquitination and degradation of GPX4	↓	[28]
	β-elemene promotes lysosomal degradation of GPX4 via TFEB	↑	[29]
	METTL14 enhances the stability and expression of GPX4 mRNA through IGF2BP1-mediated m6A modification of GPX4	↓	[30]
	Lapatinib inhibits the expression of GPX4	↑	[31]
	Notch3 inhibits the expression of GPX4	↑	[32]
NRF2	Trabectedin inhibits the KEAP1/NRF2/GPX4 axis	↑	[35]
	NSUN2 enhances the stability of NRF2 mRNA by modifying its 5′UTR region with m5C	↓	[36]
	NRF2 upregulates PHKG2 expression, induces mitochondrial dysfunction, and promotes ferritinophagy	↑	[37]
	APOC1 regulates the NRF2/HO-1 pathway	↑	[38]
	NRF2 through the FOCAD-FAK signaling pathway regulates the sensitivity of NSCLC cells to cystine deprivation-induced ferroptosis	↑	[39]
p53	Disulfide/copper through ferroptosis enhances the therapeutic efficacy of adavosertib in p53-deficient NSCLC	↑	[44]
	Qingrehuoxue formula promotes the expression of p53	↑	[45]
	KIAA1429 upregulates the p53 signaling pathway	↑	[46]
	HEATR1 upregulates the p53/SAT1/ALOX15 pathway	↑	[47]

Note: ↑, increase; ↓, decrease.

## 5. Therapeutic Strategies Targeting Ferroptosis in NSCLC

Although the existing studies have shown some improvement in the treatment of NSCLC, the clinical outcomes are still unsatisfactory. Growing evidence suggested that ferroptosis inducers held great potential as a therapeutic target for NSCLC [49]. The application of gene therapy, natural drugs, chemical compounds, nanoparticles, and ferroptosis inducers, either alone or in combination, has attracted increasing attention from researchers. Combining these strategies may enhance therapeutic efficacy and provide additional treatment options for NSCLC patients.

### 5.1. Non-Coding RNA

It is well established that the majority of the human genome was transcribed to RNAs that do not encode proteins, including long non-coding RNAs (lncRNAs), microRNAs (miRNAs), and circRNAs, which played central roles in regulating the initiation and progression of various cancers [50]. Accumulating studies have demonstrated that non-coding RNAs are the key regulators of ferroptosis, offering potential novel therapeutic strategies for NSCLC [51,52,53] (Table 3).

#### 5.1.1. miRNAs

miRNAs possess great potential as diagnostic biomarkers for NSCLC and various other types of cancer [54,55]. MiR-744-5p/miR-615-3p and miR-324-3p have been reported to reverse cisplatin resistance and inhibit tumorigenesis in NSCLC by prompting GPX4-mediated ferroptosis [56,57]. The overexpression of miR-27a-3p reduced erastin-induced ferroptosis in NSCLC cells by directly targeting SLC7A11 [58]. The natural compound sanggenol L was found to sensitize NSCLC to ferroptosis by upregulating miR-26a-1-3p, which silenced the expression of the E3 ubiquitin ligase MDM2, resulting in the downregulation of p53-mediated SLC7A11 protein expression [59]. Additionally, ionizing radiation induced the expression of miR-139 through the transcription factor EGR1, which directly targeted cJUN, disrupting NRF2 signaling and increasing the radiosensitivity of NSCLC cells [60]. MiR-302a-3p suppressed tumorigenesis by directly targeting the 3′UTR of ferritin, reducing its protein expression and inducing ferroptosis in NSCLC [61]. Therefore, the regulation of ferroptosis by miRNAs plays a crucial role in NSCLC therapy and contributes to reducing drug resistance in NSCLC patients.

#### 5.1.2. LncRNAs

A growing number of studies suggested that lncRNAs influenced cancer progression by regulating ferroptosis [62]. It is reported that LncRNA ITGB2-AS1 enhanced cisplatin resistance in NSCLC by suppressing p53-mediated ferroptosis through the activation of the FOS2/NAMPT axis [63]. The RGMB-AS1-NAA10 and RGMB-AS1-HO-1 axes were found to synergistically inhibit the growth of NSCLC cells by promoting ferroptosis both in vitro and in vivo. Mechanistically, RGMB-AS1 interacted with HO-1 to enhance HO-1 stability by blocking the E3 ligase TRC8-mediated ubiquitination [64]. LncRNA BBOX1-AS1 modulated tumorigenesis and ferroptosis in NSCLC by post-transcriptionally regulating prominin 2 expression via the sponge miR-326 [65]. Additionally, lncRNA LUCAT1 contributed to ferroptosis by downregulating GTP cyclohyrolase 1 via microRNA-34a-5p in NSCLC [66]. Xu et al. [67] demonstrated that β-elemene enhanced the sensitivity of EGFR-mutated NSCLC to erlotinib by upregulating lncRNA H9 to induce ferroptosis. Moreover, SETD1A was proposed to inhibit ferroptosis by mediating H3K4me3 methylation to upregulate lncRNA HOXC-AS3 expression and increase EP300 stability in NSCLC cells [68]. However, the potential mechanisms of lncRNAs mediating ferroptosis in NSCLC remain to be further investigated.

#### 5.1.3. circRNAs

Emerging studies have demonstrated that circRNAs were involved in various cellular and biochemical processes and played a crucial role in the therapy of NSCLC [69]. CircSCN8A was found to promote ferroptosis while inhibiting cell proliferation and metastasis in NSCLC by upregulating ACSL4 expression through the modulation of miR-1290 [53]. Other studies have shown that silencing Circ_0082374 and CircDTL induced ferroptosis in NSCLC cells through the miR-1287-5p/GPX4 or miR-491-5p/GPX4 axis, providing a novel therapeutic strategy for NSCLC patients [70,71]. Knockdown CircPDSS1 inversely regulated the SLC7A11/GPX4/GCLC axis by upregulating miR-137, promoting ferroptosis rather than other forms of cell death in NSCLC [72]. Additionally, CircPOLA2 suppressed tumorigenesis by interacting with merlin, a key regulator of the Hippo pathway, leading to the activation of the Hippo pathway and enhancing ferroptosis in NSCLC cells [73].

In summary, the regulation of ferroptosis by non-coding RNAs represents a highly promising frontier in NSCLC therapy. The integration of the miRNA, lncRNA, and circRNA-mediated pathways provides a multifaceted approach to overcoming critical challenges, including drug resistance, tumor progression, and chemoresistance, ultimately facilitating the development of more effective and personalized treatment strategies. Although a total of 16 RNA-based drugs have received clinical approval, RNA-based anticancer agents remain in Phase II/III clinical trials. The targeted delivery of ncRNAs to tumor cells still faces several challenges. These include their diverse molecular targets, as well as inherent physicochemical limitations such as large molecular weight and negative charge, which hinder cellular uptake. Additionally, LncRNAs are susceptible to rapid degradation by plasma and tissue RNases upon systemic administration [74]. Therefore, the development of efficient and targeted delivery systems is essential to ensure that ncRNA drugs can successfully enter tumor cells and exert their therapeutic effects.

**Table 3:** Therapeutic mechanisms of non-coding RNA in regulating ferroptosis for NSCLC.

Category	Target Protein/Pathway	Model	Mechanism	References
miR-744-5p/miR-615-3p	GPX4	A549, H1299, A549, and H12999 (Cis)	miR-744-5p/miR-615-3p upregulation: ↓GPX4.	[56]
miR-324-3p	GPX4	A549/DPP	miR-324-3p upregulation: ↓GSH, ↓GPX4, and ↓mitochondrial shrinkage and mitochondrial ridge; ↑ROS, ↑lipid peroxides, and ↑sensitivity to cisplatin.	[57]
miR-27a-3p	SLC7A11	A549, Beas-2B, And Calu-3; clinical tissue specimens	miR-27a-3p upregulation: ↓SLC7A11; ↑Fe^2+^, ↑ROS, ↑MDA, and ↑lipid peroxidation.	[58]
miR-26a-1-3p	MDM2	A549, H1975, and BEAS-2B; xenotransplantation of tumor in mice; patient-derived tumor xenograft (PDX)	miR-26a-1-3p upregulation: ↓SLC7A11, ↓GSH, ↓GPX4, ↓MDM2, and ↓mitochondrial contraction; ↑ROS, ↑lipid peroxides, and ↑p53.	[59]
miR-139	cJUN, KPNA2	A549, H1299, and 293FT; xenotransplantation of tumor in mice	miR-139 upregulation: ↓NRF2, ↓KPNA2, ↓SLC7A11, ↓GCLC, ↓GPX4, ↓TXNRD1, ↓HO-1, and ↓NQO-1.	[60]
miR-302a-3p	FPN	A549, H358, H1299, H1650, BEAS-2B, and HBE	miR-302a-3p upregulation: ↓GSH, ↓GPX4, and ↓cell viability; ↑Fe^2+^ and ↑lipid peroxides.	[61]
LncRNA ITGB2-AS1	FOSL2/NAMPT pathway	BEAS-2B, SK-MES-1, NCI-H520, A549, H197, and PC-9; xenotransplantation of tumor in mice	ITGB2-AS1 downregulation: ↓p53; ↑Fe^2+^ and ↑cisplatin resistance.	[63]
LncRNA RGMB-AS1	HO-1, NAA10	293T, A549, H1299, HCC827, and PC9; xenotransplantation of tumor in mice	RGMB-AS1 upregulation: ↓GSH, ↓GPX4, ↓HO-1 ubiquitination, and ↓mitochondrial shrinkage and mitochondrial ridge; ↑Fe^2+^, ↑lipid ROS, ↑MDA, and ↑NAA10 activity.	[64]
BBOX1-AS1	miR-326, PROM2	BEAS-2B, A549, H1299, SK-MES-1, and NCI-H520; xenotransplantation of tumor in mice	BBOX1-AS1 downregulation: ↓GSH, ↓GPX4, ↓SLC7A11, and ↓PROM2; ↑ACSL4 and ↑MDA.	[65]
LncRNA LUCAT1	miR-34a-5p, GCH1	BEAS-2B, A549, 293T, and H460	LUCAT1 downregulation: ↓GCH1; ↑miR-34a-5p and ↑lipid peroxidation.	[66]
LncRNA H19		BEAS-2B, H1975, H1650, and H1819(EGFR-mutant); xenotransplantation of tumor in mice	H19 upregulation: ↓GSH, ↓GPX4, and ↓MMP; ↑ROS, ↑lipid ROS, ↑MDA, and ↑cytotoxicity.	[67]
LncRNA HOXC-AS3	SETD1A, EP300	BEAS-2B, H522, PC9, H1975, and A549 xenotransplantation of tumor in mice	HOXC-AS3 upregulation: ↓SETD1A, ↓ROS, and ↓MDA; ↑GPX4, ↑GSH, ↑EP300, and ↑cell proliferation.	[68]
CircSCN8A	miR-1290	BEAS-2B, A549, H1299, NCI-H520, and SK-MES-1; xenotransplantation of tumor in mice	CircSCN8A upregulation: ↓GSH and ↓cell proliferation and metastasis; ↑ROS, ↑MDA, ↑Fe^2+^, and ↑ACSL4.	[53]
Circ_0082374	miR-491-5p	16HBE, H1299, Calu-6, and A549; clinical tissue specimens	Circ_0082374 downregulation: ↓GPX4, ↓cell proliferation and metastasis, and ↓tumor volume and weight; ↑miR-491-5p.	[70]
CirCDTL	miR-1287-5p	BEAS-2B, H23, H522, PC9, and A549; xenotransplantation of tumor in mice	CirCDTL downregulation: ↓GSH, ↓GPX4, and ↓tumor volume and weight; ↑miR-1287-5p, ↑Fe^2+^, ↑ROS, ↑lipid peroxides, and ↑sensitivity to chemotherapeutic/ferroptosis inducing agent.	[71]
CircPDSS1	miR-137/SLC7A11	16HBE, PC-9, NCI-H522, A549, and H1299	CircPDSS1 downregulation: ↓SLC7A11, ↓GPX4, ↓GCLC, and ↓cell proliferation; ↑miR-137 and ↑lipid peroxides.	[72]
CircPOLA2	Merlin-YAP	A549 and PC9	CircPOLA2 upregulation: ↓p-Merlin (Ser518), activated the hippo signaling pathway, and ↓cell proliferation; ↑MDA and ↑lipid peroxides.	[73]

Note: ↑, increase; ↓, decrease.

### 5.2. Natural Compounds

In recent years, despite significant advancements in NSCLC therapy, major challenges remain due to drug toxicity and cellular resistance. The emergence of natural compounds has attracted considerable attention due to their unique structural features and extensive biological activity. A growing number of studies have demonstrated that natural compounds have the potential to serve as NSCLC therapeutic drugs by regulating ferroptosis. Therefore, this review provides a comprehensive summary of the potential applications of various natural compounds in NSCLC therapy through the modulation of ferroptosis (detailed information is available in Table 4).

#### 5.2.1. Terpenoids

Numerous studies have demonstrated that terpenoids possessed anti-tumor properties in various cancers and were closely associated with ferroptosis in NSCLC. Li et al. [75] proposed that androhelide suppressed the proliferation and metastasis of NSCLC by inducing ferroptosis through mitochondrial dysfunction, which is characterized by the depolarization of MMP, higher levels of ROS, and lower levels of ATP. The combination of β-elemene and erlotinib enhanced the toxicity of primary EGFR-TKI-resistant NSCLC cells with EGFR mutation via H19-induced ferroptosis [67]. Additionally, the TFEB-mediated lysosomal degradation of GPX4 contributed to β-ELE-induced ferroptosis in NSCLC, providing a novel therapeutic strategy for NSCLC [29]. Wang et al. [76] discovered that 13-oxyingenol dodecanoate derivatives as a potential anti-tumor drug in NSCLC by targeting TMBIM to trigger mitophagy and ferroptosis. Meanwhile, cucurbitacin B induced ferroptosis in NSCLC by targeting and inhibiting STAT3 phosphorylation with a drop in mitochondrial membrane potential (MMP) depolarization [77].

#### 5.2.2. Alkaloid

As a natural compound, alkaloid presents promising possibilities for conversion into therapeutic drugs for NSCLC. It has been shown that Sinapine treatment resulted in the upregulation of TF/TFR and the p-53-dependent downregulation of SLC7A11, initiating ferroptosis in NSCLC cells [78]. Capsaicin has been proposed as a potential anticancer agent by inactivating the SLC7A11/GPX4 pathway, suppressing NSCLC cells’ proliferation, and inducing ferroptosis [79]. Additionally, the combination of berberine with multiple ferroptosis inducers synergistically suppressed NSCLC by depleting GSH via the p53-dependent SLC7A11-GPX4 pathway, accompanied by the aberrant accumulation of ROS and MDA [80]. Xu et al. [81] found that sanguinarine, a natural benzophenidine alkaloid, suppressed NSCLC growth and metastasis by triggering ferroptosis through STUB1-mediated ubiquitination of endogenous GPX4. Fascaplysin was demonstrated to exert anticancer effects both in vivo and in vitro by promoting apoptosis and ferroptosis, enhancing PD-L1 expression to improve sensitivity for anti-PD-1 immunotherapy [82].

#### 5.2.3. Flavonoids

Recent studies have investigated the interaction of flavonoids with NSCLC in regulating ferroptosis through multiple mechanisms. Sanggenol L was reported to sensitize NSCLC cells to ferroptosis by upregulating miR-26a-1-3p, which silenced the expression of the E3 ubiquitin ligase MDM2, resulting in the downregulation of p53-mediated SLC7A11 protein expression [59]. The synergistic effect of ginkgetin and cisplatin (DDP) inactivated the NRF2/HO-1 axis, enhancing ferroptosis in NSCLC by downregulating SLC7A11 and GPX4 expression, promoting the anticancer effects of cisplatin [83]. Additionally, Chen et al. [84] found that treatment with S-3′-hydroxy-7′, 2′, 4′-trimethoxyisoxane induced ferroptosis in NSCLC cells by inhibiting the NRF2/HO-1 pathway.

#### 5.2.4. Others

Timosaponin AIII has been found to induce the ferroptosis-mediated inhibition of proliferation and migration in NSCLC cells by targeting SP90, promoting the ubiquitination and degradation of GPX4 [85]. Another study suggested that α-Hederin reversed cisplatin resistance in NSCLC by silencing SLC7A11 and inhibiting miR-96-5p, activating DDIT3/ATF3-mediated ferroptosis [86]. Additionally, Bufotalin is emerging as a potential anti-tumor agent targeting ferroptosis by promoting the ubiquitination of GPX4 and increasing the intracellular Fe^2+^ level in NSCLC cells [87]. Curcumin enhanced ferroptosis in NSCLC by activating autophagy, which involved in mitochondrial membrane rupture, reduction in mitochondrial integrity, and an increase in autophagy markers such as LC3 and Beclin1, alongside a decrease in P62 [88]. Moreover, HO-3867, a curcumin analog, exerted anti-tumor activity by inducing ferroptosis in NSCLC cells through the activation of the p53/DMT1 axis, which inhibited GPX4 and regulated autophagy markers [89].

These insights reflect the growing recognition of natural compounds as promising therapeutic agents for the treatment of NSCLC through the modulation of ferroptosis. However, the mechanisms of action vary significantly among different classes of compounds. For instance, many alkaloids and related compounds influence ferroptosis primarily by regulating GPX4 activity, whereas flavonoids predominantly act through modulation of the NRF2/HO1 signaling pathway. These findings further underscore the central roles of GPX4 and the NRF2/HO1 axis in ferroptosis regulation, highlighting the need for deeper investigation to identify the most efficacious compounds and their key mechanisms of action. Furthermore, the multitarget properties of natural compounds make them attractive candidates for drug development, but they may also pose risks of off-target toxicity. Therefore, future research should focus on bridging the gap between basic science and clinical application by optimizing compound screening and mechanism elucidation through rigorous experimental design and integrative methodologies.

**Table 4:** Therapeutic effects of natural compounds on ferroptosis in NSCLC.

Medicine	Class	Model	Mechanism	References
Andrographolide	Terpenoids	H460 and H1650; xenotransplantation of tumor in mice	↑ROS, ↑MDA, ↑Fe^2+^, ↑lipid peroxides, ↑ATP, and ↑mitochondrial membrane potential depolarization; ↓GSH, ↓GPX4, and ↓SLC7A11.	[75]
ß-Elemene	Terpenoids	BEAS-2B, H1975, H1650, and H1819 (EGFR-mutated EGFR-TKI-resistant NSCLC cells); xenotransplantation of tumor in mice	↑lncRNA H19 and ↑ROS; ↓SLC7A11, ↓GSH, ↓GPX4, ↓FTH1, and ↓NRF2.	[67]
ß-Elemene	Terpenoids	A549TFEB KO and A549WT; orthotopic NSCLC NOD/SCID mice	↑Fe^2+^ and ↑lipid peroxides; ↓GPX4.	[29]
13-oxyingenol dodecanoate	Terpenoids	BEAS-2B, A549, H460, and LO2	↑NRF2-KEAP1-HO-1, ↑lip ROS, ↑MDA, ↑lipid peroxides, and ↑mitophagy; ↓X_c_^−^, ↓GSH, and ↓GPX4.	[76]
Cucurbitacin B	Terpenoids	H358, A549, H23, H1650, and PC9	↑Fe^2+^, ↑ROS, ↑MDA, and ↑lipid peroxides; ↓p-STAT3, ↓GSH, and ↓MMP.	[77]
Sinapine	Alkaloid	A549, SK, H661, HBE, H460, H460 and p53; xenotransplantation of tumor in mice	↑TF, ↑TFR1, ↑P53, ↑Fe^2+^, and ↑ROS; ↓SLC7A11.	[78]
Capsaicin	Alkaloid	A549 and NCI-H23	↑Fe^2+^; ↓SLC7A11, ↓GSH, and ↓GPX4.	[79]
Berberine	Alkaloid	A549, NCI-H1299, NCI-H1975, 293 T, and LLC-1; xenotransplantation of tumor in mice	↑ROS; ↓NRF2, ↓SLC7A11, ↓GSH, and ↓GPX4.	[80]
Sanguinarine	Alkaloid	A549 and H3122; xenotransplantation of tumor in mice	↑Fe^2+^, ↑ROS, and ↑MDA; ↓GSH and ↓GPX4.	[81]
Fascaplysin	Alkaloid	A549; syngeneic mouse	↑Fe^2+^, ↑ROS, and ↑PD-L1; G1/G0 phase arrest.	[82]
Sanggenol L	Flavonoids (Isopentenyl)	A549, H1975, and BEAS-2B; xenotransplantation of tumor in mice patient-derived tumor xenograft (PDX);	↑miR-26a-1-3p, ↑p53, ↑lipid peroxides, and ↑ROS; ↓MDM2, ↓SLC7A11, ↓GPX4, ↓GSH, and ↓mitochondrial contraction.	[59]
Ginkgetin	Flavonoids (Bioflavonoid)	A549, NCI-H460, and SPC-A-1; xenotransplantation of tumor in mice	↑MMP; ↓NRF2/HO-1, ↓SLC7A11, ↓GSH/GSSG, and ↓GPX4.	[83]
S-3′-hydroxy-7′, 2′, 4′-trimethoxyisoxane	Flavonoids (Isoflavonoid)s	A549 and H460; xenotransplantation of nude mice	↑Fe^2+^, ↑ROS, and ↑MDA; ↓GSH, ↓NRF2/HO-1, ↓GPX4, and cell cycle arrest.	[84]
Timosaponin AIII	Saponin	H1299, A549, SPC-A1, Lewis lung carcinoma(LLC), and HBE; xenotransplantation of tumor in mice	↑ROS, ↑MDA, And ↑HSP90; ↓GSH and ↓GPX4.	[85]
α-Hederin	Saponin	A549/DPP and PC-9/DPP; xenotransplantation of tumor in mice	↑EGR1 and ↑ DDIT3/ATF3; ↓miR-96-5p, ↓SLC7A11, and ↓GPX4.	[86]
Bufotalin	Steroids	A549; xenotransplantation of tumor in mice	↓GPX4	[87]
Curcumin	Polyphenol	A549 and H1299; Lewis lung carcinoma mice	↑Fe^2+^, ↑ACSL4, ↑lipid peroxides, ↑LC3, ↑Beclin1, and ↑autolysosomes; ↓SLC7A11, ↓GSH, ↓GPX4, ↓P62, and ↓mitochondrial cristae.	[88]
HO-3867	polyketide	H460, PC-9, H1975, A549, H1299, A549 p53, and H460 p53 KO	↑Fe^2+^, ↑ROS, and ↑DMT1 in a p53-dependent way; ↓GPX4.	[89]

Note: ↑, increase; ↓, decrease.

### 5.3. Combination Therapy

**Figure 3:**
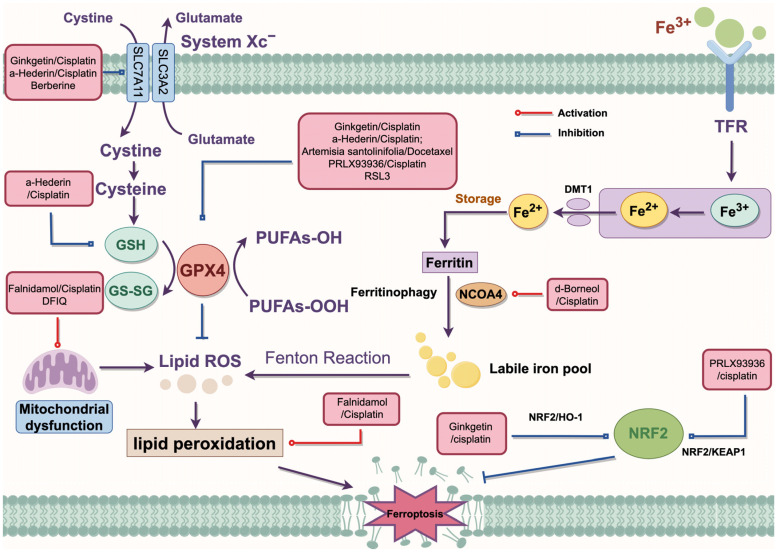
The regulatory mechanism of combination therapy. Artemisia santolinifolia/Docetaxel inhibits GPX4 to enhance DTX-induced cell death. RL3 inhibits the expression of GPX4 to promote ferroptosis. α-Hederin/cisplatin inhibits SLC7A11 and GPX4 to activate ferroptosis. In addition, α-Hederin/cisplatin disrupts the redox process to inhibit GSH synthesis. Ginkgetin/cisplatin inhibits the expression of SLC7A11 and GPX4 through the NRF2/HO-1 axis to promote cisplatin sensitivity. Berberine acts with multiple inducers to consume GSH through the SLC7A11-GPX4 pathway. Falnidamol/cisplatin and DFIQ induce mitochondrial dysfunction. d-Borneol/cisplatin promotes NCOA4-mediated ferroptosis. PRLX93936/cisplatin silences the NRF2/KEAP1 axis to promote ferroptosis in NSCLC cells. DMT1, divalent metal transporter 1; GPX4, glutathione peroxidase 4; GSH, glutathione; HO-1, heme oxygenase 1; KEAP1, Kelch-like ECH-associated protein; NRF2, Nuclear factor erythroid 2-related factor 2; NCOA4, Nuclear receptor coactivator 4; ROS, reactive oxygen species; System X_c_^−^, cystine/glutamate antiporter system; SLC3A2, solute carrier family 3 member 2; SLC7A11, solute carrier family 7 member a11; TFR, transferrin receptor.

In recent years, traditional medicines have shown great potential in cancer therapy. It has been reported that 178 small molecules mediated the process of ferroptosis [90]. Currently, the chemotherapy drugs used clinically for NSCLC can be categorized into the following classes: (1) alkylating agents (such as cisplatin); (2) microtubule-targeting drugs (such as docetaxel, and vinorelbine); (3) antimetabolites (such as pemetrexed and gemcitabine); (4) topoisomerase inhibitors that block topoisomerase enzymes (such as etoposide) [91]. Combination therapies have been shown to enhance the sensitivity of NSCLC cells to chemotherapy through multiple pathways. The specific mechanisms are summarized in Figure 3.

#### 5.3.1. The Combination of Natural and Chemical Medicines

It is well established that ginkgetin promoted the anticancer effects of cisplatin by inactivating the NRF2/HO-1 axis and enhancing ferroptosis through the downregulation of SLC7A11 and GPX4 expression in NSCLC [83]. The combination of d-menthol and cisplatin-induced ferroptosis by promoting NCOA4-mediated ferritinophagy and upregulating intracellular iron transport. Furthermore, d-menthol combined with cisplatin promoted autophagy by upregulating the expression of LC3II/ATG5/Beclin-1, while inhibiting epithelial–mesenchymal transition (EMT), thereby enhancing cisplatin sensitivity in NSCLC [92]. α-Hederin has been found to reverse cisplatin resistance in NSCLC by silencing SLC7A11 and inhibiting miR-96-5p, activating DDIT3/ATF3-mediated ferroptosis [86]. Additionally, α-Hederin treatment inhibited GSH synthesis and disrupted the GSH redox system by downregulating the expression of glutathione peroxidase 2 (GPX2) and glutathione synthetase (GSS), thereby inducing ferroptosis at a low-toxicity dose and enhancing cisplatin chemotherapy resistance in NSCLC cells [93]. Falnidamol combined with cisplatin was demonstrated to enhance the cytotoxicity of cisplatin and inhibit NSCLC cells’ proliferation by downregulating the expression of dual-specificity phosphatase 26 (DUSP26), leading to ferroptosis through free iron accumulation and lipid peroxidation [94]. Another study has suggested that Artemisia santolinifolia ethanol extract synergistically enhanced the cytotoxicity of DTX against NSCLC cells in a caspase-dependent manner by inhibiting the STAT3/Survivin signaling pathway, promoting ferroptosis through GPX4 downregulation and ROS accumulation [95].

#### 5.3.2. Anti-Tumor Therapy Combined with Ferroptosis Inducers

PRLX93936, an analog of ferroptosis inducer erastin, has been tested in two clinical phase I/II trials for various advanced cancers (NCT00528047) and multiple myeloma (NCT01695590). Liang et al. [96] discovered that PRLX93936 synergized with cisplatin-induced ferroptosis in NSCLC cells by incrementing ROS, peroxidation, and Fe^2+^ levels, while reducing GPX4 expression through the NRF2/KEAP1 pathway. RSL3 has also been demonstrated to induce ferroptosis by suppressing GPX4 expression via activation of the NRF2/HO1 pathway in NSCLC cells [97]. DFIQ sensitized NSCLC cells to sorafenib by inducing ferroptosis through autophagic dysfunction and mitochondrial damage [98]. Li et al. [99] demonstrated that the ferroptosis inducers erastin and sorafenib effectively triggered ferroptosis in N5CP cells by modulating the expression of NRF2 or xCT, leading to excessive accumulation of intracellular lipid ROS, thereby altering the sensitivity of NSCLC cells to CDDP. The combination of berberine with multiple ferroptosis inducers, including sulfasalazine (SAS), RSL3, FIN56, and dihydroartemisinin (DHA), synergistically suppressed NSCLC by depleting GSH via the p53-dependent SLC7A11-GPX4 pathway. This effect was accompanied by the aberrant accumulation of ROS and MDA [80].

These results highlight the potential of combination therapy as an innovative strategy to enhance ferroptosis in NSCLC, offering the possibility of improving chemotherapy efficacy and overcoming the current therapeutic limitations. However, with the exception of PRLX93936, most combination strategies remain limited to preclinical studies and lack supporting clinical trial data. Moreover, their multitarget mechanisms may result in unforeseen toxicities or the emergence of drug tolerance. Overall, while the existing strategies demonstrate the capability of combination therapy to induce ferroptosis in NSCLC, critical challenges such as toxicity management, elucidation of resistance mechanisms, and resolution of clinical translation barriers must be systematically addressed.

### 5.4. Nanomaterials

Recently, the development of nanodrugs has provided promising insights for ferroptosis-based anti-tumor therapies. Nanomaterials preferentially accumulate in tumor tissues due to the enhanced permeability and retention effect, offering advantages such as high drug-loading capacity, prolonged drug release duration, and reduced side effects [100]. Currently, the rare nanomaterials that target ferroptosis for non-small cell lung cancer treatment are summarized in Table 5.

VF/S/A@CaP is a nanocatalytic sensitizer that delivers vitamin C, si-OTUB2, and ASO-MALAT1 to efficiently prevent NSCLC growth and metastasis by blocking the interaction between amyloid precursor protein and death receptor 6. Furthermore, the Fe^2+^ released by Vc-Fe(II) generated cytotoxic hydroxyl radicals (•OH) via the Fenton reaction, resulting in ferroptosis and overcoming Osimertinib (AZD9291) resistance in NSCLC cells [101]. Fe_3_O_4_-TA-GOD/anti-LILRB4&Sor@MSN-DMMA (FTG/L&SMD) has also been demonstrated to enhance ferroptosis by initiating the Fenton reaction for NSCLC therapy. Mechanistically, iron ions convert the H_2_O_2_ generated by glucose oxidase on FTG/L&SMD into •OH via the Fenton reaction, resulting in the accumulation of lipid peroxidation [102]. In recent years, Tian et al. [103] reported that albumin-encapsulated Pt(IV) nanodrug (HSA@Pt(IV)) activated ferroptosis by disrupting iron homeostasis in NSCLC cells. Folic acid-modified liposome nanoparticle (E/M@FA-LPs) stimulated erastin-induced ferroptosis by increasing intracellular lipid ROS and depleting GSH through the miR-365a-3p/NRF2 axis [104]. Additionally, Tsai et al. [105] found that CD44-hyaluronic acid (HA)-mediated endocytosis could be utilized to deliver iron–platinum alloy nanoparticles (FePt NPs), inducing ferroptosis in mesenchymal-state lung cancer cells and overcoming tyrosine kinase inhibitor (TKI) resistance. Kaushik et al. [106] developed PLGA-based nanoparticles (CPBA − PLGA(SOR + SIM) − NPs) modified with 4-carboxyphenylboronic acid (CPBA), encapsulating sorafenib (SOR) and simvastatin (SIM). It significantly reduced intracellular GSH levels, increased ROS and MDA production, and induced mitochondrial membrane depolarization, collectively triggering ferroptosis-mediated anticancer effects in A549 cells. Moreover, Zero-valent iron nanoparticles (ZVI-NPs) have been reported to exhibit a dual anticancer mechanism that spares nonmalignant cells. On one hand, it promoted GSK3β/β-TrCP-dependent NRF2 degradation by activating the AMPK/mTOR signaling pathway, thereby selectively triggering ferroptosis in lung cancer cells. On the other hand, it activated anti-immunogenic responses by reprogramming tumor-associated macrophages from the protumorigenic M2 phenotype to the anti-tumor M1 phenotype, increasing the cytolytic activity of CD8⁺ T lymphocytes as well, while reducing the proportion of Treg cells [107]. Meanwhile, Hu et al. [108] developed chitosan-based nanoparticles (CNPs) by coating the amino-crosslinked shell of superparamagnetic iron oxide nanoparticles (CLIO-NH2, CN) with N-isopropylacrylamide (PEN), which were further functionalized with small interfering Snail2 RNA (siSnail2) to form CNP@siSnail2 nanoparticles. CNP@siSnail2 promoted both apoptosis and ferroptosis in A549 cells through multiple mechanisms, effectively inhibiting EMT, as well as the migration and invasion of cancer cells. Notably, the combination of CNP@siSnail2 and cisplatin exhibited a synergistic tumor-suppressive effect. In addition, FP@SFN, a multifunctional nano-formulation consisting of silk fibroin-based nanocarrier (SFN) co-loaded with FIN56 and piperlongumine (PPL), was designed to enhance cytotoxicity against lung cancer cells. This nanoplatform exerted a dose-dependent anti-tumor effect through the synergistic enhancement of ferroptosis and autophagy driven by oxidative stress [109].

In conclusion, the advent of nanodrugs in ferroptosis-based therapies represents a significant advancement in the treatment of NSCLC. By harnessing the unique properties of nanomaterials, these therapies can improve the efficacy of ferroptosis induction, circumvent drug resistance, and provide more targeted and precise treatment options for NSCLC patients. However, despite the significant potential of nanodrugs in ferroptosis-based anti-tumor therapy, their clinical application remains limited due to several challenges. In contrast to the extensive body of preclinical research, only a few nanomaterials-based drugs have been successfully translated into clinical practice. To bridge this gap, future efforts should prioritize minimizing toxicity, enhancing delivery efficiency and stability, and clarifying the mechanisms underlying the enhanced permeability and retention effect in humans. Additionally, the safety and long-term efficacy of nanomaterials systems such as VF/S/A@CaP, FTG/L&SMD, and HSA@Pt(IV) require more comprehensive validation in diverse in vivo models.

### 5.5. CRISPR-Cas9 Technology

The development of gene-editing technologies based on DNA nucleases has progressed rapidly. Advancements from the first-generation zinc finger nucleases (ZFNs) to the second-generation transcription activator-like effector nucleases (TALENs), and subsequently to the third-generation CRISPR systems have led to continuous improvements in editing efficiency and specificity, along with an expanded range of applications. Among these, the CRISPR-Cas9 system has been widely utilized in various studies due to its early discovery, simple structure, and ease of modification [110]. The advent of CRISPR-Cas9 technology has introduced a powerful and precise gene-editing tool for targeting and overcoming drug resistance mechanisms in lung cancer. CRISPR-Cas9 enables the precise addition, deletion, or modification of genes, thereby disrupting key drug-resistance pathways and re-sensitizing cancer cells to the existing therapies [111]. In recent years, relatively rare but noteworthy studies have applied CRISPR-Cas9 technology to the targeted regulation of ferroptosis in NSCLC (Table 6).

CRISPR-Cas9 screening identified miR-6077 as a key driver of cisplatin/pemetrexed (CDDP/PEM) resistance in LUAD. The overexpression of miR-6077 desensitized LUAD cells to CDDP/PEM in both cell lines and in patient-derived xenograft models. Mechanistically, miR-6077 protected LUAD cells from cell death induced by CDDP/PEM via CDKN1A-CDK1-mediated cell-cycle arrest and ferroptosis mediated by the KEAP1-NRF2-SLC7A11/NQO1 axis. These effects collectively contributed to multidrug resistance in LUAD, highlighting miR-6077 as a potential therapeutic target to overcome chemoresistance in clinical practice [112]. Moreover, pooled CRISPR screens have identified MAFF as a tumor suppressor in LUAD. Clinically, decreased MAFF expression was associated with poor prognosis in LUAD patients. Functional studies demonstrated that MAFF enhanced LUAD cell sensitivity to cisplatin and ionizing radiation by inducing ferroptosis through the downregulation of SLC7A11. These findings suggest that the pharmacological activation of MAFF could potentially improve the efficacy of cisplatin or radiotherapy in LUAD treatment [113]. In addition, TFEB knockout cells were generated using the CRISPR/Cas9 genome editing system. TFEB deficiency was found to impair the β-elemene-induced lysosomal degradation of GPX4, thereby suppressing the anticancer effects of β-elemene through the inhibition of ferroptosis in an orthotopic NSCLC model [29]. Similarly, the CRISPR-mediated knockout of the TFEB in LUAD cells reduced K48-linked ubiquitination and inhibited TRIM25-mediated GPX4 lysosomal degradation, consequently attenuating ginkgetin-induced ferroptosis in EGFR wild-type LUAD [114]. Overall, these results indicate that TFEB is a potential therapeutic target to enhance ferroptosis-mediated cancer treatment in NSCLC.

Recent advances in CRISPR-Cas9 technology have enabled the precise targeting of key genes and regulators involved in ferroptosis, thereby enhancing the sensitivity of NSCLC cells to ferroptosis. By focusing on critical nodes such as GPX4, KEAP1, TFEB, and MAFF, CRISPR-based strategies have revealed complex interactions among redox homeostasis, lysosomal degradation, and therapeutic responsiveness. These findings highlight the potential of CRISPR not only as a gene-correction tool but also as a platform for developing personalized ferroptosis-based therapeutic strategies in lung cancer. Nevertheless, most of these studies have not evaluated the long-term stability of gene-edited cell lines during extended culture or experimentation, nor have they investigated the potential compensatory expression or functional redundancy of related genes following the knockout of targets such as TFEB or GPX4. Furthermore, concerns regarding off-target effects, biosafety, and ethical considerations continue to pose significant challenges for the clinical translation of CRISPR-Cas9 technology [115].

The therapeutic potential of CRISPR-Cas9 technology in NSCLC relies heavily on the precise delivery of its components into target cells, as well as the development of strategies that minimize off-target effects. Yang et al. [116] engineered a CRISPR-dCas9 system to activate PTEN expression, thereby restoring the therapeutic efficacy of gefitinib in NSCLC. To enhance target specificity and delivery efficiency, gefitinib and the dCas9 system were co-loaded onto mesoporous silica nanoparticles (MSNs) through physical encapsulation and electrostatic adsorption. Finally, a nano-delivery system, M-G-P/P@CM, was formed by mechanical extrusion coating with tumor cell membrane (CCM), significantly improving the sensitivity of NSCLC patients to gefitinib. These findings highlight the potential of integrating CRISPR-based tools with advanced delivery systems to overcome drug resistance, offering a promising strategy for personalized and effective therapy in NSCLC. Moreover, it provides valuable insights into future precision-targeting strategies designed to enhance ferroptosis susceptibility in NSCLC cells.

## 6. Conclusions and Perspectives

**Figure 4:**
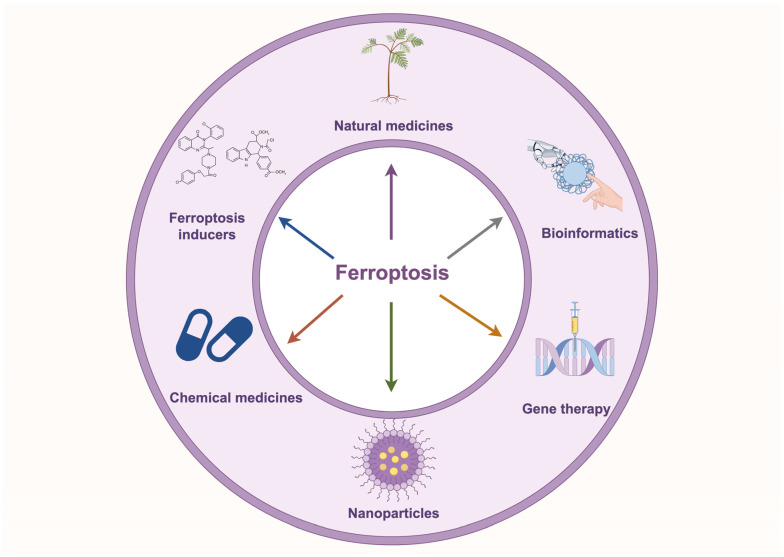
Application of ferroptosis-targeted therapies in NSCLC. The diagram illustrates ferroptosis-based targeted therapeutic strategies for NSCLC, including natural compounds, ferroptosis inducers, chemical drugs, nanomaterials, gene therapy, and bioinformatics approaches, providing novel directions for NSCLC treatment.

Ferroptosis is closely associated with tumorigenesis and drug resistance in NSCLC. Numerous studies have demonstrated that modulating ferroptosis directly influences tumorigenesis and drug resistance in NSCLC by altering iron metabolism, lipid metabolism, and amino acid metabolism. Non-coding RNAs play a critical role in regulating the sensitivity of NSCLC cells to ferroptosis by modulating the expression of key ferroptosis-related genes. Moreover, emerging therapeutic strategies, including natural compounds, ferroptosis inducer-based combination therapies, nanomaterial-based delivery systems, and CRISPR-mediated gene editing, are showing promise in inducing ferroptosis, offering new directions for NSCLC treatment (Figure 4).

Despite the promising therapeutic potential of ferroptosis in NSCLC, there are still several limitations. First, the molecular mechanisms underlying ferroptosis are highly complex and not yet fully elucidated. Current studies have identified key regulators such as SLC7A11, NRF2, and p53, which modulate ferroptosis in NSCLC primarily through the regulation of GPX4, suggesting that GPX4 may be a key effector for ferroptosis. Moreover, p53 has been shown to influence ferroptosis by regulating the expression of SLC7A11, GPX4, and NRF2, positioning it as a potential upstream regulator for ferroptosis. However, these targets also play important roles in regulating ferroptosis across various other tumor types. Lee et al. [26] reported that ferroptosis inducers, natural compounds and their derivatives, medicinal compounds, and repurposed drugs can directly inactivate or degrade GPX4. This mechanism effectively triggered ferroptosis and suppressed the progression of cancers such as bladder cancer, breast cancer, neuroblastoma, and gastric cancer. Additionally, gankyrin and tanshinone IIA have been shown to inhibit ferroptosis in triple-negative breast cancer [117] and gastric cancer [118] through the modulation of the p53/SLC7A11 axis, respectively. Moreover, few studies focus on the investigation of the differential sensitivity of various NSCLC subtypes to ferroptosis or the verification of related biomarkers. It is essential to identify NSCLC subtypes that are specifically regulated by ferroptosis and to characterize key biomarkers that can guide the development of ferroptosis-based targeted therapies. Therefore, comparative analyses of ferroptosis sensitivity among different NSCLC subtypes, along with the identification of key biomarkers, are essential for guiding ferroptosis-based targeted therapeutic strategies in NSCLC.

In addition, natural compounds and ferroptosis inducers have shown considerable promise, especially in combination therapies aimed at overcoming resistance in NSCLC. However, only a limited number, such as PRLX93936, have progressed to clinical trials. One major challenge is that natural products often consist of complex mixtures with diverse chemical structures and compositions, which complicates the evaluation of their pharmacological activities and tissue specificity. These complexities further hinder the accurate assessment of their absorption, distribution, metabolism, and excretion (ADME) properties. Moreover, ferroptosis has been found to be closely associated with neurodegenerative diseases, cardiovascular disorders, and other pathological conditions [119], suggesting that the induction of ferroptosis in NSCLC may inadvertently trigger or aggravate other pathological processes due to its non-specific targeting.

Despite these challenges, nanomedicines and CRISPR technology hold considerable promise for advancing ferroptosis-based targeted therapies in NSCLC. Future research should prioritize the optimization of delivery systems, the enhancement of gene-editing specificity, and a thorough investigation of compensatory mechanisms that may arise following genetic modification. The in-depth exploration of specific regulatory targets involved in ferroptosis, identification of key biomarkers across NSCLC subtypes, and integration of tumor microenvironmental factors are crucial for achieving precision therapeutic interventions. The application of advanced CRISPR and nano-delivery platforms may enable highly specific induction of ferroptosis in NSCLC cells, thereby facilitating more effective and personalized treatment strategies. Furthermore, comprehensive studies on pharmacodynamics and toxicology, along with rigorous clinical validation, are essential to ensure the safety and efficacy of these emerging therapies and to promote their successful clinical translation in NSCLC therapy.

## Figures and Tables

**Table 1:** Comparative morphological and biochemical characteristics of ferroptosis, apoptosis, necroptosis, and autophagy.

Characteristics	Ferroptosis	Apoptosis	Necroptosis	Autophagy
Morphological	Loss of cell membrane integrity; shrinkage of mitochondria, increased mitochondrial membrane density, and reduction in mitochondrial cristae morphology; and chromatin condensation	Intact cell membrane; reduced cell volume with organelles, maintaining normal morphology; condensation of the nucleus and cytoplasm; and fragmentation of the nucleus	Rupture of the cell membrane; swelling of mitochondria; and irregular condensation of nuclear chromatin	Intact cell membrane; presence of autophagosomes, autolysosomes, and vacuoles; and nucleus: absence of chromatin condensation
Biochemical	↑iron, ↑ROS, and ↑lipid peroxidation; ↓GSH.	↑caspases, internucleosomal DNA fragmentation.	↑GSDMD, ↑NLRP3, ↑IL-1β, and ↑IL-18.	↑ratio of LC3-II/I; ↓p62.
Key regulatory factors	↑p53 and ↑TFR1; ↓GPX4, ↓SLC7A11, and ↓ACSL4.	↑Caspase-3, ↑Caspase-8, and ↑BAX; ↓BCL-2.	↑Caspase-1, ↑TNF-α, ↑RIP1, ↑RIP3, and ↑MLKL.	↑AMPK, ↑ULK1, ↑ATG, and ↑BECN1; ↓mTOR.

Note: ↑, increase; ↓, decrease.

**Table 5:** Application of nanomaterials in ferroptosis-based anti-tumor therapies for NSCLC.

Nanoparticle Name	Nanoparticle Size	Model	Mechanism	References
VF/S/A@CaP	150 nm	AZD9291; tumor growth on cell-derived xenograft (CDX)	↑Fenton reaction; ↓GPX4, ↓GSH, OTUB2 and MALAT1 downregulation, cell invasion, and metastasis.	[101]
FTG/L&SMD	350 nm	A549; xenotransplantation of tumor in mice	↑Fenton reaction, ↑ROS, and ↑cytotoxicity; ↓GPX4, ↓VEGF, and ↓tumor migration and angiogenesis.	[102]
HSA@Pt(IV)	49.5 nm	A549, H1299, and MRC-5; Homologous and xenotransplantation of tumor in mice	↑lipid ROS and ↑PTGS2 mRNA; ↓GSH and mitochondria damage.	[103]
E/M@FA-LPs	154 nm	A549 and H1299	↑MT1DP, ↑ROS, ↑lipid ROS, and ↑MDA; ↓NRF2 and ↓GSH.	[104]
FePt NPs	6 nm	HCC827, GR+, and M6; xenotransplantation of tumor in mice	↑EMT, ↑P62, ↑ NRF2, ↑DHODH,↑SLC7A11, ↑PTGS2, ↑TFR1, ↑HO-1, ↑FTH1, ↑Fenton reaction, ↑lipid ROS, and ↑lipid peroxides; ↓KEAP1 and ↓GPX4.	[105]
CPBA − PLGA(SOR + SIM) − NPs	213.1 ± 10.9 nm	A549; BALB/c mouse model	↑ROS and ↑MDA; ↓GSH and mitochondrial membrane depolarization.	[106]
ZVI-NP	ZVI@Ag NPs: 81.08 ± 14.29 nm; ZVI@CMC NPs: 70.17 ± 14.4 nm.	H1299, H460, A549, MRC-5, IMR-90, and LLC; xenotransplantation of tumor in mice	↑AMPK/mTOR, loss of mitochondrial membrane potential, ↑mtROS, ↑Fenton reaction, ↑oxidative stress, and ↑lipid peroxides; ↓NADPH, ↓GPX4, and disrupt the AMPK/mTOR pathway to activate p-GSK3/β-TrCP and in turn degrade NRF2, ↓PD-1, and CTLA4 in CD8+ T cells.	[107]
CNP@siSnail2	60 nm	A549; subcutaneous tumor model and tail vein injection model	↑ROS; ↓GSH and ↓migration and invasion.	[108]
FP@SFN	193 ± 5.86 nm	A549; subcutaneous tumor	↑ROS and ↑lPO; ↓GPX4, ↓Q10, ↓GSH, and loss of mitochondrial membrane potential.	[109]

Note: ↑, increase; ↓, decrease.

**Table 6:** Application of CRISPR-Cas9 technology for the targeted regulation of ferroptosis in NSCLC.

Function	Model	Mechanism	References
Screening system reveals miR-6077 as a key regulator of CDDP/PEM sensitivity in LUAD	A549,H358, and HEK293T; xenotransplantation of tumor in mice	miR-6077 overexpression: ↓CDDP/PEM resistance and ↓ROS; ↑GSH, ↑NRF2, ↑SLC7A11, and ↑NQO1.	[112]
Screening identifies MAFF as a tumor suppressor in LUAD	A549, A549/DDP, H358, HCC827, PC-9, H1975, BEAS-2B, and HEK293T; xenotransplantation of tumor in mice	↓SLC7A11, ↓glutamate, ↓GSH, ↓mitochondrial membrane density, and ↓mitochondrial cristae.	[113]
Knockout TFEB	A549^TFEB KO^ cells; mouse model	TFEB knockout: ↓lysosomal degradation of GPX4 and ↓anticancer effect	[29]
Knockout TFEB	A549^TFEB KO^ cells (TFEB-/--2, TFEB-/--4); xenotransplantation of SCID mouse models	TFEB knockout: ↓lysosomal activity, ↓cathepsin B, ↓GPX4-TRIM25 binding force, ↓LIP, and ↓lipid peroxides; ↑GPX4.	[114]

Note: ↑, increase; ↓, decrease.

## Data Availability

Not applicable.

## Abbreviations

The following abbreviations are used in this manuscript:

ACSL4	Acyl-CoA synthetase long-chain family member 4
ALKBH5	AlkB homolog 5
ALOX	arachidonate lipoxygenase
ATP	Adenosine-5′-triphosphate
ATF3	Three-letter acronym
DDIT3	DNA damage-induced transcriptional activator 3
DMT1	divalent metal transporter 1
EGR1	Early growth response 1
EP300	E1A binding protein p300
FPN	Ferroportin
FSP1	Ferroptosis-suppressor protein 1
FTH1	Ferritin heavy chain 1
CAP	Cold atmospheric plasma
GCH1	GTP cyclohydrolase 1
GCLC	glutamate/cysteine ligase catalytic subunit
GPX4	glutathione peroxidase 4
GSSG	oxidized glutathione
GSH	glutathione
GSR	glutathionedisulfide reductase
GSS	glutathione synthetase
γ-GCS	Gamma-glutamylcysteine synthetase
HO-1	heme oxygenase 1
HSP90	Heat shock protein 90
KEAP1	Kelch-like ECH-associated protein 1
KPNA2	Karyopherin subunit alpha 2
LAPTM4B	lysosome-associated protein transmembrane 4B
LC3	microtubule-associated protein 1A/1B-light chain 3
LPCAT3	lysophosphatidylcholine acyltransferase 3
MDA	Malondialdehyde
MDM2	Mouse double minute 2 homolog
MLKL	Mixed lineage kinase domain-like pseudokinase
MMP	mitochondrial membrane potential
NAA10	N-α-acetyltransferase 10
NCOA4	Nuclear receptor coactivator 4
NQO-1	NAD(P)H:quinone oxidoreductase 1
NRF2	Nuclear factor erythroid 2-related factor 2
PD-L1	Programmed death-ligand 1
PROM2	prominin 2
PPAR	peroxisome proliferator-activated receptor
PPRE	peroxisome proliferator response element
PTGS2	Prostaglandin–endoperoxide synthase 2
P62	Sequestosome-1
Q10	Coenzyme Q10
RIP1	Receptor-interacting serine/threonine-protein kinase 1
RIP3	Receptor-interacting serine/threonine kinase 3
ROS	reactive oxygen species
SETD1A	SET domain containing 1A
SLC3A2	solute carrier family 3 member 2
SLC7A11	solute carrier family 7 member 11
SPTBN2	Non-Erythrocytic 2
System X_c_^−^	cystine/glutamate antiporter system
TF	transferrin
TFR	transferrin receptor
TRIM3	tripartite motif-containing 3
TXNRD1	Thioredoxin reductase 1
